# Citation Network Analysis on the Influence of Vision on Academic Performance

**DOI:** 10.3390/children10030591

**Published:** 2023-03-20

**Authors:** Sandrina Esteves, Clara Martinez-Perez, Cristina Alvarez-Peregrina, Miguel Ángel Sánchez-Tena

**Affiliations:** 1 ISEC LISBOA (Instituto Superior de Educação e Ciências), 1750-142 Lisbon, Portugal; 2 Optometry and Vision Department, Faculty of Optics and Optometry, Complutense University of Madrid, 28037 Madrid, Spain

**Keywords:** childhood, learning, vision

## Abstract

Background: Proper vision is absolutely critical to children’s academic performance, as vision problems can drastically affect learning ability. Currently, the existing literature in this field is somewhat inconsistent and carries several controversies about the influence of vision on academic performance. In this study, citation networks were utilized in order to analyze the relationship between publications and authors, the most-cited publication, and the different research areas. Additionally, the most commonly utilized publication sources along with the most common research areas were also pinpointed. Methods: The aforementioned search was executed in the *Web of Science* database, with a time range beginning in 1941 and ending in 2022. In order to scrutinize the publications, VOSviewer, CiteSpace software, and the Citation Network Explorer were utilized for analysis about the most-cited publication and the different research areas. Results: Overall, 1342 papers were found in all fields along with 2187 citation networks. Moreover, 2020 was the year with the most publications, including 127 publications and 4 citation networks. Bull et al., published in 2008, was the most-cited work, reaching a citation index of 975. The clustering function managed to identity four groups with the most engaging research topics from researchers: motor visual skills, visual memory, the influence of vision on the different learning styles, and abnormal development of the visual system. Conclusions: In the end, the topic with the greatest interest was the influence of visual motor skills on academic performance. Ideally, this paper will assist fellow researchers in quickly noting which topics are of greatest interest and how research in this area has evolved.

## 1. Introduction

Students’ school success is among the most researched and studied socio-educational variables among authors. As a matter of fact, it has become a greatly pressing problem in the education politics and government discourse in many nations around the world. Places such as the United States of America, the United Kingdom, South Korea, Iceland, Sweden, Norway, Belgium, the Netherlands, Austria, and Germany have all been grappling with how to approach education successfully [1].

School success goes far beyond the grade earned at the end of a semester or school year. Thus, success in this area has to be seen from an integrated view, and this success can be achieved by pupils in diverse areas [1]. Many factors play a role in determining school success, especially the political and social complexities. Thus, there is a requirement to pay attention to a nation’s social policies involving education and, likewise, the socio-cultural framework of the students attending the schools and their family background. Everything counts: the institution’s curriculum and framework, the management, the teachers and pupils, the available resources, and the environment and pedagogical options. (This means the dispositions, attitudes, and behaviors of both the students as a group and also specifically of each student [1].)

Azevedo (2013) argued that there is a complex set of dimensions that directly influences school success, namely (i) an individual dimension directly related to variables such as students’ gender/ethnicity, cultural and family environment, personal relationships network, behavior and attendance, and attitude towards school; (ii) a pedagogical–didactic dimension, which is made up of variables such as classroom management, teacher/student relationship, curricular organization, teaching and learning methodologies, and assessment practices; (iii) an institutional dimension with variables associated with the school environment, the school educational project, the type of management and leadership of the school, the involvement of parents towards the school, and the school’s own educational policy; and (iv) a community, family, and local dimension with variables directly dependent on the involvement of the local community on education, level of education of the population, and the standard of living in the municipality [2]. In addition, also regarding these dimensions, there is the very functioning of the education system, particularly with regards to educational policy options, as well as issues such as the age of compulsory minimum education, models for funding schools, education and curricular guidance of the school, or even the system for hiring teachers. The academic performance in varying levels of education is a key matter in several local, national, and international institutions. For instance, the Organization for Economic Co-operation and Development (OECD) Program for International Student Assessment (PISA 2019 report) ensures the improvement of students’ performances by intaking proposals from governments worldwide [3]. They do so because, as noted earlier, performance is influenced by multiple factors [4]. Thus, governments must develop effective policies to improve all aspects that might have a negative impact on learning; for example, health at school age frequently affects educational aspirations [5]. Within health, optical health is critical, and poor vision, which can be due to either genetic or outside causes, is a perfect example. A person suffering from poor vision has a visual acuity over 0.5 (standard logarithmic scale), whereas a normal visual acuity would be approximately 0.00 or less [6]. Poor eyesight is considered both a chronic and progressive disability, one which can adversely impact students’ scholastic performance, educational potential, crucial movement skills, physical fitness, and mental health [7,8]. Recently, it was discovered that all school-aged children are especially vulnerable to this issue [9]. This is somewhat unsurprising, given the frequency of myopia [10,11]. Maples [12] suggested that sight-related risk factors tend to predict academic performance even more than socioeconomic status and race. This is because visual perception allows us to interpret the information that surrounds us through the visible light that reaches the eye [13]. Visual disturbances vary depending on their origin and severity. Therefore, they range from simple refractive errors to binocular problems and visual impairment. These visual deficiencies can produce a wide variety of symptoms that affect learning abilities [14]. Reduced vision can lead to low motivation for the exploration of the information that surrounds us, minor social interactions, and manipulation of objects [15]. Thus, students with poor vision cannot share visual experiences with their classmates, and therefore, the loss of vision can have a negative impact on the development of social skills [15,16,17]. Up to 40% of school-aged children may have vision problems that affect their visual function [18]. However, certain visual abnormalities may go unnoticed in traditional screening techniques, which are mainly based on the measurement of visual acuity of high contrast and binocular tests [19]. Therefore, visual–motor integration, visual movements, visual memory, processing speed, and visual search correspond to reading ability and other areas of academic performance such as arithmetic [20,21,22,23,24]. This fact is very important due to the role vision plays in school and academic performance [25,26,27]. Moreover, educational achievement influences long-term social, economic, and health outcomes [1].

A large number of studies, both epidemiological and educational, have confirmed the ubiquity of poor vision at school age [28,29,30,31,32,33]. This is mostly caused by time spent performing tasks that require constant strain on the eyes and also due to their sedentary lifestyles. Students spend over 70% of school time performing visually demanding academic tasks that also require sustained focus [34]. Unfortunately, students who spend more time reading, writing, and studying naturally also carry a larger risk of developing vision problems compared to their other classmates [35,36]. On the other hand, the female sex is also a risk factor, with various studies showing a higher prevalence of myopia amongst females [11,37]. It is possible that, in general, females may be more inclined to participate in close-up reading activities in lieu of physical activities such as sports, or they are simply less encouraged to do so [38]. Due to the vast pool of information, multiple research papers have been developed and have elaborated on the influence of vision on academic performance. Different databases such as the *Web of Science*, *ISI*, *Scopus*, and *Google Scholar* organize the information to utilize computer programs that manage a massive amount of data and organize, store, publish, distribute, and treat many studies. The citation networks analysis for this paper was carried out using Citespace and VOSviewer.

With a calculated examination of citation networks, we can analyze the composition of a research field. Despite being around for some time, this is still new in the field of vision and its effects on education. In this analysis, the goal is to assist researchers in more easily grasping these complex scientific structures. Hopefully, this analysis allows the reader to gain relevant and modern information within this field. It is primarily meant for new researchers and will highlight the most-prominent authors and publications alongside other ones. Additionally, this will help form a chronological order of study and add to the research over time, offering information on conceptual issues.

In summary, a citation network analysis allows us to find additional relevant publications for the sake of qualitatively and quantitatively displaying the connections between articles and authors by creating groups [39]. Furthermore, it is possible to quantify the most-cited publications within each of these said groups [40,41].

The aim of this study was to identify the different research areas and determine the most-cited publication through the analysis of citation networks on the importance of vision in academic performance. This identification is based on the fact that the citation of a work is an authoritative acknowledgment of a previous work. Most of the bibliometric indicators that measure scientific impact (impact factor, H index, crown indicator, etc.) have citation as a central element of their analysis. For all these reasons, the analysis of the citation network gives us the bibliometric value of the publications.

## 2. Materials and Methods

### 2.1. Database

The search for publications was carried out in the *Web of Science* (*WoS*) database. *WoS* was chosen since it only hosts journals with a worldwide presence after they successfully pass a meticulous selection process. It also hosts imperative information on bibliometric analysis and citation networks. This means it includes author affiliations, the number of citations, the title, the abstract, and the keywords. These items might not typically be present in other similar databases [42].

The following search terms were used: “academic performance OR academic achievement” AND “vision OR eye OR visual”. The selected time interval for the search was from 1941 to November 2022. The publications were searched and downloaded on 16 November 2022 in the “topic” field. The provided information had the number of publications and their year of publication, the affiliations of the authors and institutions, the citations per year and most-cited articles, and lastly, the keywords and abstracts.

### 2.2. Data Analysis

The Citation Network Explorer (Ness Jan van Eck and Ludo Waltman, Centre for Science and Technology Studies (CWTS), Leiden University, Leiden, The Netherlands) software was used to analyze the publications and obtain citation networks. Once the analysis was completed, it became feasible to manage millions of interconnected publications and citations [41].

Subsequently, the most-connected publications were assigned to the same group using the clustering function. The clustering function allows the grouping of previously unconnected publications. Because of this, the most-connected ones typically wind up within the same group [41]. The publications considered to be the core of a citation network were analyzed using the Identifying Core Publications functionality. In this analysis, only those publications with four or more citations were considered. The software established the minimum number of connections, and in doing so, the publications marked as less prominent were removed [40]. Additionally, the drilling down functionality was used to analyze the different levels of each group.

Besides this, the CiteSpace software (5.6.R2; Chaomei Chen, College of Computing and Informatics, Drexel University, Philadelphia, PA, USA) was used to perform scientometric analysis by establishing certain parameter indicators, with the “H index” used to evaluate the quantity and level of academic output of researchers and academic institutions [43]. The “degree” parameter indicated the number of connections among authors, institutions, and countries in the co-occurrence knowledge graph; consequently, a higher value in this degree indicated that there was a greater level of communication and collaboration. On top of this, the “intermediate centrality” parameter decides the research cooperation network. The parameter notes the number of times a node acts as a waypoint along the shortest link between the two other nodes, which is called the “geodesic distance”. The “Half-life” parameter is responsible for deciding the continuity of institutional research from a time perspective. If a citation peak is reached, which is denoted by a low value, then it will decline rapidly. Likewise, a high value that is exceeded once the maximum point of citation has been reached instead decreases slowly [44].

## 3. Results

The first articles highlighting the relationship between academic performance and a student’s vision were published in 1941. Therefore, the time interval selected to perform the search was from 1941 to December 2021.

Nevertheless, it is important to highlight that in recent years, especially in the last 11 years, there has been an exponential increase in papers on school success and vision (1941–2009: 23.4%; 2010–2021: 76.6%).

**Figure 1:**
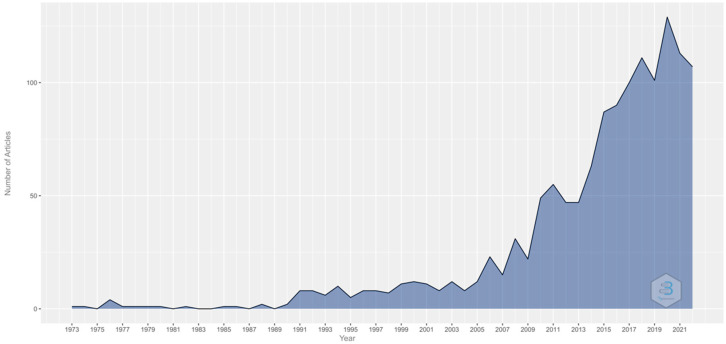
Number of publications per year.

In total, 1342 publications were found in all areas along with 2187 citation networks after the bibliography search was undertaken. Further, 2020 was the year with the most publications, including 127 publications and 4 citation networks (Figure 1).

Publications overview

Of all the publications, 79.1% were made up of papers, 14.9% were proceeding papers, 5% were review papers, and 1% were chapters of books.

With regards to the language of the publications, 94.4% were in English, 2.7% were in Spanish, and 0.6% were in German.

**Figure 2:**
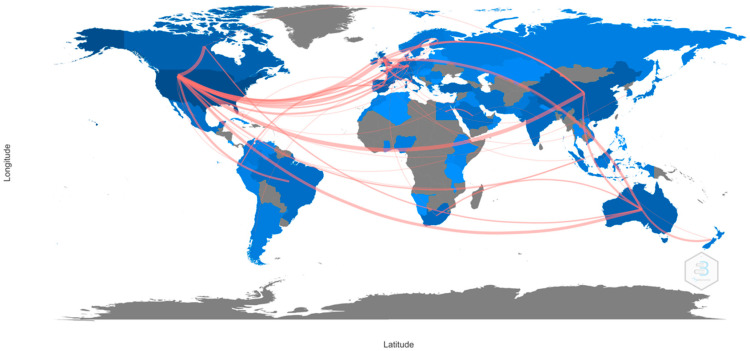
Connection amongst countries.

As shown in Figure 2, the countries with the highest number of publications and connections with other countries were the United States (publications: 436; degree: 37; connections: 358), Spain (publications: 79; degree: 15; connections 51), and China (publications: 72; degree: 9; connections: 123).

Education/educational research (69.7%), and psychology (22.9%) had the greatest number of publications by far (Table 1).

Table 2 and Table 3 display the ten authors and the ten institutions with the highest number of publications. The three authors with the highest number of publications were Pienaar AE (publications: 0.5%; degree: 0; connections: 4), Wood JM (publications: 0.5%; degree: 5; connections: 24), and Calhoun SL (publications: 0.4%; degree: 1; connections: 8). Likewise, the three institutions with the highest number of publications were the University of California System (2.9%), Harvard University (2.6%), and the League of European Research Universities Leru (2.6%).

Table 4 displays the primary journals and the number of publications in each of these journals. Thus, it was observed that the three journals with the highest number of publications were *Frontiers in Psychology* (Switzerland) with 24 publications; *Perceptual and Motor Skills* (United States) with 14 publications; and *PloS one* (the United States) with 13 publications.

**Figure 3:**
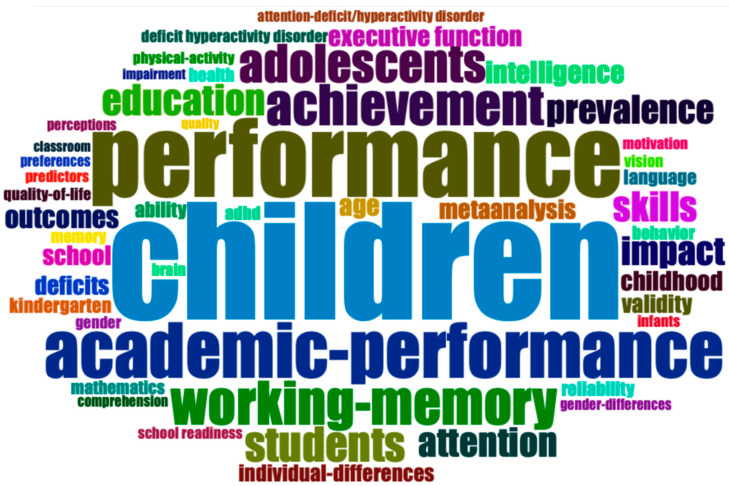
Word cloud based on the most commonly used keywords.

Additionally, the most commonly used keywords were “Children”, “Academic-Achievement”, and “Performance” (Figure 3). On the other hand, Table 5 shows in more detail the frequency at which each word was used. Thus, from the 20 most-used keywords, the word “Children” was used 195 times, “Academic-Achievement” 154 times, and the word “Performance” 131 times.

Table 6 showcases the most-cited publications. As can be seen, Bull et al. [45] has the highest number of citations. The aim of this study was to analyze whether short-term memory, working memory, and executive skills in children are predictors of their academic performance. Research showed that short-term visuospatial memory capacity was a specific predictor of mathematical ability. The correlational and regression analysis showed, in turn, that short-term visual and working memory specifically predict mathematics performance, whereas skills related to executive function are predictors of learning in general as opposed to learning in a specific domain.

Clustering

The clustering function identified five groups with the most-engaging research topics, of which four had a significant number of publications. The remaining group corresponded only to 0.5% of publications.

**Figure 4:**
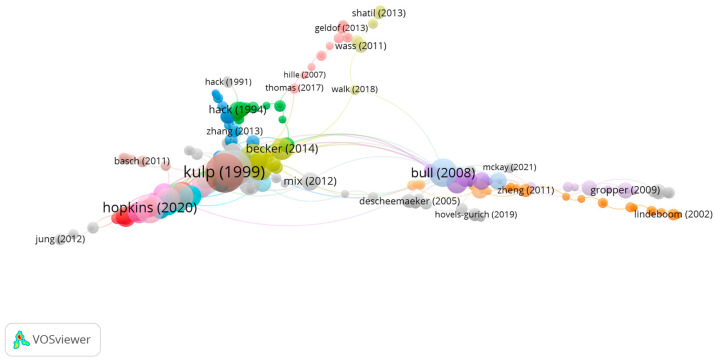
Citation network in group 1.

In group 1, there were 157 publications and 373 citations (Figure 4). The most-cited publication was the article by Kulp et al. [21], which was published in 1999 in *Optometry and Vision Science.* The paper written by Kulp et al. aimed to analyze the relationship between visual–motor integration skill and academic performance during infancy. Therefore, the Beery-Buktenica Developmental Test of Visual–Motor Integration was used since it was confirmed that both were significantly related to academic performance in children aged 7, 8, and 9 years of age.

The articles in this group analyzed the influence of motor–visual skills on academic performance.

Group 2 is made up of 54 publications and 68 citation networks. The publication by Bull et al. [45], in 2008, in *Developmental Neuropsychology* is the one with the highest number of citations. This was also the most-cited publication out of the 20 most-cited publications.

**Figure 5:**
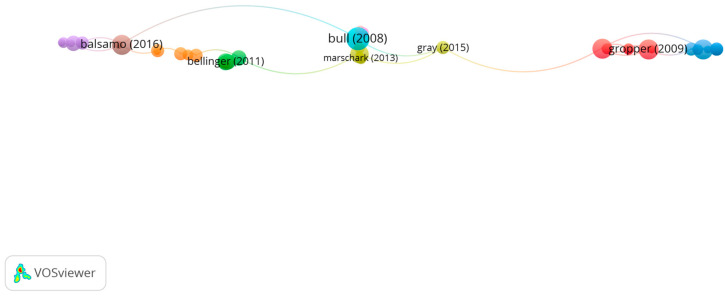
Citation network in group 2.

The articles in this group analyze the influence of visual memory on academic performance (Figure 5).

Group 3 is made up of 39 publications and 71 citation networks. The publication by Nuzhat et al. [55], in 2013, in *Medical Teacher*, is the one with the highest number of citations. In this study, the Visual, Aural, Read/Write, and Kinesthetic (VARK) questionnaire, version 7.0, was used on medical students. The aim was to know the difference in learning styles, according to gender and the effect it has on academic performance. It was observed that most of the students preferred a multimodal learning style. These students had higher scores when compared with students who preferred a unimodal learning style.

**Figure 6:**
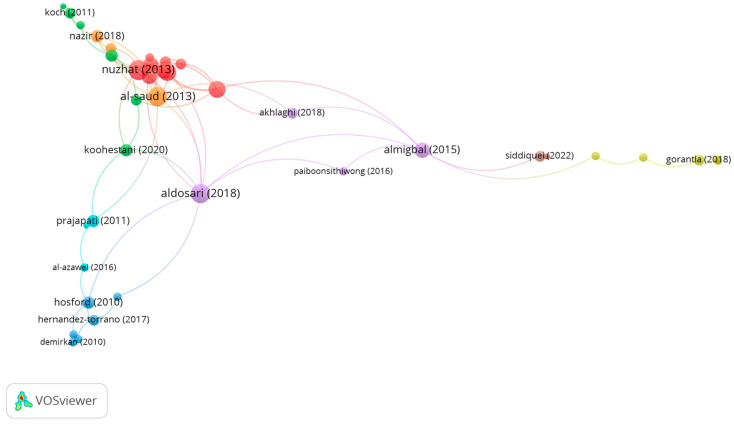
Citation network in group 3.

The publications in this group analyze the influence of vision on the different learning styles (Figure 6).

Group 4 is made up of 13 publications and 16 citation networks. The most-cited publication was the one by Hack et al. [47], which was published in 1994 in *The New England Journal of Medicine*. This study compared academic performance in premature children (on the one hand children weighing less than 750 g and on the other hand children weighing 750 g to 1499 g) with full-term children. Growth, neurosensory status, and school-aged functioning were compared in the three groups. It was observed that the children weighing less than 750 g presented a higher risk of neurobehavioral dysfunction and a poor school performance.

**Figure 7:**
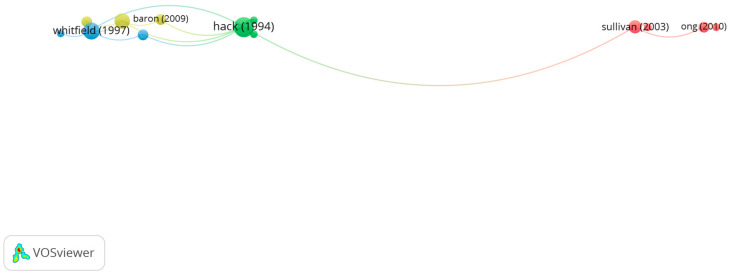
Citation network in group 4.

The articles in this group analyze the ways in which the lack of development of the visual system and the higher risk to develop pathologies in premature children relate to academic performance (Figure 7).

## 4. Discussion

The aim of this study was to conduct a bibliometric and citation network analysis on the influence of vision on academic performance. Thus, this study provided simple and visual information to offer a better understanding of different aspects of vision in academic performance. This type of study is new to this field of research, so it will help researchers to have a better understanding of underlying scientific structures. That is to say, it establishes for the reader the bases of the knowledge that currently exists based on the investigations with the greatest interest from other researchers. Primarily, it helps new researchers to find the most-cited research texts, prominent authors, and sources in order to find the fundamental research that shapes a field of research. At the same time, it also allows them to understand the historical lineage of outstanding research over time and offers information on the main topics to show how it has progressed over time and also the steps followed by researchers.

It was found that the number of publications has increased significantly since 2010. This matches the study by Tao et al. [56], in which they conducted a bibliometric analysis on academic procrastination and its relationship with academic performance; they found that since 2013, the number of publications in this field of research has increased rapidly. Thus, in the study by Tao et al. [56], in 2007, the number of publications was 31; in 2013, it was 53; and in 2020, it was 111. This shows the interest on the part of researchers to analyze how various factors influence academic performance. The consistent interest in the factors that influence academic performance may possibly be because of the high dropout rate, primarily in college. In relation to this interest, Basch et al. [57] published, in 2011, a study carried out in the United States, concluding that it did mattered neither how well-prepared teachers were, nor government measures, nor student motivation if health was impaired. Furthermore, 2021 was the year with the highest number of publications. In this year, the publication by Liu et al. [58], in which they assessed how visuospatial ability predicts academic performance in Chinese students through arithmetic and reading skills, stands out. Thus, after analyzing 490 children, they showed that visuospatial ability has a direct impact on the academic performance of primary school students as measured by arithmetic and reading skills. This is consistent with the fact that it is one of the topics with the most interest on the part of researchers.

With regards to collaboration between countries, the United States is the country with the highest number of publications. It also has strong scientific collaboration and exchange in this field of research with the United Kingdom, China, Canada, and Australia. This can be related to the fact that the United States has a long tradition of research and spatial practice in the form of education. In 1975, the Education for All Act was passed, which, since 2004, has been known as the Individuals with Disabilities Education Act [59]. In turn, its strong relationship with Australia was due to the fact that in 2008, the Australian government created the National Disability Strategy to ensure that all children with a disability can have the opportunity to participate socially and economically in the cultural life of the nation [60]. On the other hand, it is also particularly important that many authors have published high-quality and highly collaborative research with other authors and countries. For example, Nathan Congdon of Queen’s University Belfast, Orbis International, and Zhongshan Ophthalmic Center conducted a study in which they assessed the effect of wearing spectacles with the right correction on the academic performance of myopic children. The results showed that performance improved significantly in mathematics tests [61]. Harvard University and the University of California System have the largest number of publications on vision and academic performance. At Harvard University, the most-relevant publication is currently the one carried out by Rosen et al. [62], in which they demonstrated that visual processing regions influence attention processes; in turn, they provided evidence that the ventral visual stream influences academic performance. At the University of California System, Haist et al. [63] showed that two different brain systems for non-symbolic numerical acuity can contribute to individual differences in mathematical performance and that the contribution of these systems differs across development. This confirms that the United States carries massive influence thanks to its powerhouse economy and the ample amount of money and resources that can be poured into scientific research.

With regards to the journals with the highest number of publications, researchers select the journals that are most suitable for the publication of their articles. In this study, the most-active journals published only 11% of the total number of publications on vision and academic performance. This suggests that this research topic is likely to be of interest in several journals, possibly due to the diversity of research areas. Furthermore, a concordance rate of 30% was found between the ten most-active journals and the ten most-cited journals, which confirms the diversity of the journals for the publication of articles. The diversity of journals investigating the effect of vision on academic performance demonstrates it is a prevalent topic that spreads into several other areas of research. Despite this, we are firmly of the opinion that research must be centralized in a few journals in order to make things simpler to locate for all interested researchers.

With regards to the main keywords “Children”, “Academic-Achievement”, and “Performance” stand out. This is related, as shown in this study, to the fact that the main research topic is the influence of motor skills on academic performance. The publication carried out by Blanchet et al. [64] in 2022, in which they analyzed how students with specific learning disabilities may have impaired motor skills, stands out. Thus, they concluded that out of the 36 studies analyzed, all reported motor impairments, which can be seen to increase in subjects with ADHD and coordination problems. In addition, a relationship was also found between motor impairments or motor underutilization and sedentary lifestyle behavior. This could lead to health problems and therefore supports the need for comprehensive and systematic motor assessments in a young population with learning disabilities. Another study with a great relevance in this field is the one carried out by de Waal et al. [65] in the Republic of South Africa, which analyzed how various visual perception constructs can influence academic performance at school age. They concluded that basic and complex visual perceptual constructs remain important for academic performance among this age group, while gender and socio-economic status influence both visual perceptual skills and academic performance.

In the future, the most-used keywords seem to be “satisfaction”, “myopia”, and “technology” since, according to our search, they were the most used in 2021. This is related to the fact that technological advances have contributed to a change in reading habits. Reading digital texts is one of the main activities as a complement or alternative to reading books/printed material. Thus, the studies that are already analyzing how the use of technology affects academic performance has increased are nine in number since being first published in 2019.

The article by Verhoeven et al. [66], published in the year 2020, in which a meta-analysis was carried out within the last 25 years on how the use of computers for early literacy affects early childhood education, is worth noting. Technologies were found to be beneficial in early literacy. However, they must be integrated into the school curriculum and provide support for continuous instruction. In turn, it was being analyzed how the use of technology influences student satisfaction. Thus, it has been shown that the impact of technology through self-regulated learning positively contributed to academic and functional performance as well as the satisfaction of the students [67].

On the other hand, in relation to myopia, in recent years, it has been published that refractive errors influence academic performance. This is because reports of the link between factors associated with education and refractive errors among high school children have had important public health consequences. Therefore, uncorrected farsightedness has been found to be associated with lower academic performance on educational or academic achievement tests (reading ability and literacy scores).

In contrast, myopes without other binocular anomalies do not usually have near-sighted problems, unlike hypermetropes. For this reason, it has been reported that the myopic present a higher score in academic performance [68,69].

Another topic of great interest is how visual memory influences academic performance. Thus, Cockcroft [70], in a study carried out in the United States, showed that along with the growth of children, a better performance is developed. This allows a greater accumulation of knowledge and in turn a better long-term memory. In addition, the study also suggested that if the adaptive materials are adapted based on the memory skills of each student, it will help them develop more efficient learning. Recently, Dutra et al. [71] found an association between visuospatial improvement and short-term memory with respect to task resolution. These results offer an instructive perspective on the research that currently exists and a possible future direction as regards the importance of vision in academic performance. In other words, they can help researchers choose appropriate collaborators or journals to promote their research as well as knowing the main research topics of the most interest today. Our results in this study will allow future research to be conducted across distinct areas. This will surely provide better results and will assist with improving academic performance.

In the future, it is likely that research will focus on how technology influences academic performance and, consequently, its relationship with vision. This is due to the fact that the use of technology is increasingly present in schools and as a means of work and study. In turn, excessive use of technology has already been shown to negatively affect vision [72]. For this reason, more research is needed in the future to elucidate more precisely the relationship between these three factors.

Thus, this study will help future researchers to know the bases of how vision affects academic performance and how to focus their research.

## 5. Conclusions

This study provides an extensive, objective analysis on the different research fields in vision and academic performance.

It has been seen that it is an expanding area of research with growth in recent years and the possibility of sweeping new lines of research.

With the use of citation network analysis, it is plausible to state that short-term memory, working memory, and executive skills in children are predictors of their academic performance.

Furthermore, the influence of visual–motor skills on academic performance is one of the main research topics in this area of knowledge.

## 6. Implication

This study will help researchers to quickly perceive which publications carry the most interest within the different research fields regarding vision and academic performance.

Lastly, it will also help them to locate the topics of greatest interest and to carry out future research in this field.

## Figures and Tables

**Table 1:** The 10 research areas with the highest number of publications.

Category	Frequency	Centrality	Degree	Half-Life
Education/educational research	411	0.00	66	38.5
Psychology	303	0.00	52	36.5
Neurosciences neurology	130	0.00	36	21.5
Pediatrics	106	0.00	29	21.5
Computer science	85	0.00	41	13.5
Ophthalmology	79	0.00	10	29.5
Rehabilitation	63	0.00	19	32.5
Psychiatry	59	0.00	24	22.5
General internal medicine	48	0.00	11	25.5
Engineering	47	0.00	37	13.5
Business economics	39	0.00	30	9.5

**Table 2:** Top 10 authors with the highest number of publications.

Authors	Articles	Degree	Connections	Half-Life
Congdon N	7	1	346	−05
Pienaar AE	7	0	11	−0.5
Wood JM	7	5	83	3.5
Calhoun SL	6	1	-	−0.5
Coetzee D	6	-	35	-
Mayes SD	6	1	-	−0.5
Villa-Collar C	6	3	131	−0.5
Hansraj R	5	-	38	-
Hopkins S	5	2	183	−0.5
Kulp MT	5	1	197	3.5

**Table 3:** Top 10 institutions with the highest number of publications.

Category	Frequency	Centrality	Degree	Half-Life	Connections
University of California System	38	0.00	2	5.5	22
Harvard University	35	0.00	15	8.5	19
League of European Research Universities Leru	35	0.00	1	5.5	15
University of Toronto	27	0.00	6	−0.5	23
University of London	25	0.00	1	−0.5	2
Pennsylvania Commonwealth System of Higher Education (PASSHE)	23	0.00	1	−0.5	1
Hospital For Sick Children (Sickkids)	20	0.00	1	0.5	21
State University System of Florida	18	0.00	2	−0.5	4
Boston Children’s Hospital	17	0.00	2	−0.5	2
University of Texas System	17	0.00	7	3.5	1

**Table 4:** Top 10 journals with the highest number of publications.

Journal	Total Publications	Impact Factor (2021)	Quartile Score	SJR (2021)	Citations/Docs (2 Years)	Total Citations (2021)	H Index	Country
*Frontiers in Psychology*	30	4.23	Q1	0.873	3.884	38,043	133	Switzerland
*PLoS ONE*	16	3.75	Q2	0.852	3.582	188,716	367	United States
*Perceptual and Motor Skills*	15	2.21	Q3	0.498	2.371	440	73	United States
*Optometry and vision science*	10	2.11	Q3	0.561	1.747	871	102	United States
*Child Neuropsychology*	10	2.56	Q3	0.698	2.618	524	76	United Kingdom
*Journal Of Learning Disabilities*	10	3.41	Q1	1.119	3.089	380	96	The Netherlands
*Research In Developmental Disabilities*	9	3.53	Q1	0.796	2.994	1813	95	United States
*Ophthalmic And Physiological Optics*	8	3.99	Q2	1.051	3.667	672	70	United Kingdom
*Developmental Neuropsychology*	8	2.11	Q3	0.536	1.75	260	99	United Kingdom
*Developmental Medicine and Child Neurology*	7	4.86	Q2	1.398	2.773	2770	151	United States

**Table 5:** Top 20 most-used keywords.

Keyword	Frequency	Degree	Total Link Strength
Children	195	154	1338
Academic-Achievement	154	78	806
Performance	131	99	740
Academic-Performance	92	147	469
Working-Memory	73	78	500
Achievement	68	76	441
Adolescents	64	80	416
Students	58	43	324
Education	52	38	363
Skills	50	58	372
Attention	49	65	392
Impact	48	56	254
Prevalence	48	58	259
Intelligence	37	27	267
Outcomes	37	36	223
Age	34	18	195
Executive Function	34	46	246
Meta-analysis	33	32	220
School	33	40	217
Childhood	32	26	257

**Table 6:** Top 10 most-cited publications.

Paper	DOI	Total Citations
BULL R, 2008, DEV NEUROPSYCHOL [45]	10.1080/87565640801982312	975
COHEN J, 2009, TEACH COLL REC [46]	NA	734
HACK M, 1994, NEW ENGL J MED [47]	10.1056/NEJM199409223311201	515
DYRBYE LN, 2005, MAYO CLIN PROC [48]	10.4065/80.12.1613	504
BELLINGER DC, 2003, J THORAC CARDIOV SUR [49]	10.1016/S0022-5223(03)00711-6	461
HACK M, 1991, NEW ENGL J MED [50]	10.1056/NEJM199107253250403	386
THOMAS MR, 2007, J GEN INTERN MED [51]	10.1007/s11606-006-0039-6	325
BELLINGER DC, 2011, CIRCULATION [52]	10.1161/CIRCULATIONAHA.111.026963	296
BEEBE DW, 2006, SLEEP [53]	10.1093/sleep/29.9.1115	278
CORTESE S, 2015, J AM ACAD CHILD PSY [54]	10.1016/j.jaac.2014.12.010	267

## Data Availability

Not applicable.
